# The Case of Dr. Matthews

**Published:** 1907-01

**Authors:** 


### THE CASE OF DR. MATTHEWS.

Readers of this journal will recall the famous case of Dr. J. B. Matthews, of Greensboro, N. C., who was indicted, tried and sentenced to twenty years imprisonment for wife murder, the decree having been affirmed by the Supreme Court within the past few days, and still more recently the last sad chapter has been read in the newspapers—the story of the suicide of Matthews in a cheap Baltimore lodging house. Interest in the case was widespread, eminent specialists were present to testify as to the sanity of the defendant, the highest legal talent of the State battled for and against him. It is history now and we revert to it only because there has come to our table within the past week a most interesting paper by Dr. T. D. Crothers, of Hartford, Conn., who, as, an alienist and neurologist and as a totally impartial and unbiased expert, gave it as his opinion that the prisoner was, as a result of morphinism, insane and at the time when the crime was committed was wholly incompetent to reason and act normally. The writer believes, and perhaps justly, that it is an error to attempt "to administer justice and maintain the majesty of the law in the punishment of imbeciles" and that it is absurd to present to an ordinary jury the evidence of sanity and responsibility in such cases. He holds that the only wise thing to have done was to have settled definitely the question of sanity by submitting it to a commission of specialists. Insanity being admitted there could be no question of motive or responsibility. But to leave the decision of such a case in the hands of twelve men who knew absolutely nothing about the medico-legal and scientific side of the case was sheer foolishness.

We qoute herewith the main facts presented in Dr. Crother's paper:

1. The hereditary history of Matthews' case showed a distinct neurotic heritage and predisposition to mental instability, with feeble controlling power. In the ordinary strains and drains of life he was incompetent to live normally, but would most naturally develop some form of neurotic disease, according to the environment and conditions of life.

2. His defective heredity was apparent in the sensitive nervous system of early childhood and errotic conduct, manifest in suicidal mania at the beginning of manhood. This, with the history of spirit and drug taking, was the natural course of defective organism with faulty culture in bad surroundings, which would follow with absolute certainty from such conditions.

3. The fact of graduating with honors and becoming a popular physician, making and losing many friends by his errotic conduct and increasing eccentricity, was further evidence of a neuropathic condition and an unstable, unsound brain.

4. The year before the alleged crime he was notorious for his periods of stupor, depression and talkative delusional deliriums. He became careless in his dress and disregarded many of the proprieties of life, indicating great physical and mental changes. His untruthfulness, respect for his word, pride of character and petty efforts to take advantage further confirmed the fact of his defective reason and irresponsibility.

5. His relations with his wife gave no indication of any variance or disagreement, and at times in public they exhibited great respect and affection for each other. He was not considered by his friends combative or revengeful, but rather the opposite, hence there could be no motive apparent to dispose of his wife.

6. The comatose condition of his wife on the morning of her death could not be called an unusual event, where both were using morphia. His statement that she tried to commit suicide by taking a large dose of strychnine and that he had given as an antidote continuous doses of morphine every half hour, was a very natural order of events, particularly as he was using it himself. His indifference to the efforts of the physicians in restoring her and the apparent impulse to administer more morphia secretly to make the case fatal, was the act of an imbecile brain and a morbid impulse of a lunatic, without consciousness of the consequence and results of his act.

7. The further discovery of the prisoner in fail and during the trial, in which he manifested a degree of nervousness bordering on delirium, unless he was given morphia in sufficient quantities to make him comfortable. His general demented condition, errotic talk and stupor alternating with apparent sanity, but always followed by general unconsciousness concerning himself and surroundings.

From these facts there are certain general conclusions, which should have been recognized and acted upon without any possible question or doubt.

First. It was an error of the authorities in assuming that death was caused by strychnine, and not verifying it by post-mortem examination and chemical analysis of the contents of the syringe.

Second. It was a still more serious error in assuming that Matthews was sane and responsible for his acts, especially in that community, where his mental condition was a subject of common observation and talk and his drug taking was acknowledged beyond all question.

Third. The apparent motive in injecting into his wife's arm some substance to increase the fatality of her condition would naturally demand his arrest and confinement, but the assumption that this was a sane act of a responsible man was a reflection on the intelligence and judgment of the authorities.

Fourth. Matthews should have been tried by a commission of lunacy, and the questions of his mental condition determined before any further steps had been taken, then if sanity, malice and responsibility had been apparent, his trial would have been reasonable and in accord with the dictates of justice.

Fifth. Such a commission would have determined from the facts of his history, his present condition and appearance, the probable impulses, delirium or delusion which prevailed at the time of the alleged crime, and what his appearance of sanity was, as seen by the physicians before the death of his wife.—*Charlotte Med. Jour.*, Dec., 1906.

